# Gender-, Age-, and Region-Specific Associations Between Obesity and Nutrition/Health Knowledge, Dietary Diversity, and Physical Activity in Chinese School-Age Students: A Cross-Sectional Study

**DOI:** 10.3390/nu17132214

**Published:** 2025-07-03

**Authors:** Xiuwen Ren, Yu Liu, Xixiang Wang, Ronghua Li, Xiaoxiao Guo, Suhua Zhao, Rui Yan, Chi Zhang, Shaobo Zhou, Linhong Yuan, Weiwei Li

**Affiliations:** 1 Beijing Key Laboratory of Environment and Aging, China-British Joint Laboratory of Nutrition Prevention and Control of Chronic Diseases, School of Public Health, Capital Medical University, Beijing 100069, China; renxiuwen@126.com (X.R.); yu000609@163.com (Y.L.); wangxixiang0820@163.com (X.W.); 2 Jining Center for Disease Control and Prevention, Jining 272000, China; lironghua9914@163.com (R.L.); guoxx4929@163.com (X.G.); 17864711856@163.com (S.Z.); yanrui-0306@163.com (R.Y.); 3 School of Biological Sciences, University of Nebraska-Lincoln, Lincoln, NE 68583, USA; czhang5@unl.edu; 4 School of Science, Faculty of Engineering and Science, University of Greenwich, Central Avenue, Chatham ME4 4TB, UK; s.zhou@greenwich.ac.uk

**Keywords:** obesity, school-age students, dietary intake, nutritional and health literacy

## Abstract

**Background**: Chinese school-age students are at a high risk of developing obesity. However, few studies have reported individualized ways to prevent obesity by age, gender, and living regions. **Methods**: A total of 11,285 students aged 6–18 years were recruited and participated in this cross-sectional study. Questionnaires were used to assess scores of dietary diversity (DDS), physical activity (PA) duration, and nutritional literacy awareness. According to age and gender-specific BMI thresholds, the participants were categorized into normal and participants with obesity groups. Pearson correlation and logistic regression analysis were used to explore the association between nutritional literacy, DDS, PA, and the risk of BMI or obesity. **Results**: Scores of nutritional literacy were positively associated with Total DDS, Plant DDS, Animal DDS, and PA, and were negatively associated with BMI. High Total DDS (OR = 0.878, *p* = 0.030), Plant DDS (OR = 0.885, *p* = 0.027), and PA (OR = 0.869, *p* = 0.022) were strongly associated with a low risk of obesity. Furthermore, high Total DDS and Plant DDS decreased the risk of obesity only in the high PA group but not in the low PA group. High PA only decreased the risk of obesity in the high Total DDS and high Plant DDS group. Gender-, age-, and living-region-specific associations were also observed. **Conclusions**: Diverse dietary intakes and physical activity are essential for reducing the risk of obesity in Chinese school-age students. Notably, gender-, age-, and living-region-specific health and nutritional literacy education are required in school-age children to prevent obesity.

## 1. Introduction

Childhood obesity is strongly associated with high blood pressure, dyslipidemia, type 2 diabetes, and metabolic fatty liver disease [1]. As of 2021, the global prevalence of obesity stood at 93.1 million among individuals aged 5–14 years and 80.6 million among adolescents aged 15 years and older [2]. There were 159 million school-age students worldwide with obesity in 2022, a three-fold increase since 1990 [3]. Obesity constitutes a significant risk factor for metabolic syndrome, with the two conditions often coexisting and exerting reciprocal influences. The global prevalence of metabolic syndrome in 2020 was 2.8% among children aged 6–12 years and 4.8% among adolescents aged 13–18 years, corresponding to approximately 25.8 million children and 35.5 million adolescents affected by the condition [4]. In China, from 1985 to 2014, the prevalence of obesity in children and adolescents aged 7–18 years increased from 0.1% to 7.3% [5]. These data highlight the fact that obesity has become a critical public health issue and needs to be addressed urgently.

The mechanisms of childhood obesity involve complex interactions between genetic, epigenetic, and environmental factors. Genetically, it includes monogenic forms and polygenic forms. Epigenetically, transgenerational inheritance plays a role. Parental pre-conception exposures can alter gamete epigenomes, affecting offspring. In utero, maternal obesity/overnutrition, and inadequate methyl donors disrupt fetal epigenetic programming via the placenta, impacting genes related to energy metabolism and adipogenesis. Postnatally, breast milk miRNAs may also transmit epigenetic info. Environmental factors like sedentary lifestyles, high-calorie diets, and urbanization exposure interact with genetic and epigenetic susceptibilities, driving energy surplus and adipose tissue dysfunction, collectively contributing to childhood obesity [6]. Obesity can be effectively prevented through appropriate interventions. The determinants and risk factors of overweight and obesity in China can be analyzed through a three-layered framework: systemic forces, environmental drivers, and individual-level factors [7]. Unhealthy dietary habits, insufficient physical activity, and lack of health education are the primary regulatable risk factors contributing to obesity during school age [8]. The World Health Organization (WHO) recommends that children and adolescents should accumulate at least 60 min of moderate-intensity physical activity (PA) per day, on average, for a minimum of 3 days per week [9]. However, many school-age students do not meet the standard for a healthy lifestyle [10].

The Dietary Guidelines for Chinese Residents emphasize that individuals should consume more than 25 different types of foods per week [11]. The American Heart Association highlights the fact that increasing the diversity of specific food groups, such as plant foods, protein sources, low-fat dairy products, vegetable oils, and nuts, plays a crucial role in obesity prevention [12]. Physical activity plays a vital role in maintaining energy balance and promoting metabolic health. Engaging in physical activity regularly could increase energy expenditure, reduce fat accumulation, and significantly lower the risk of obesity [13]. Nutritional literacy plays a key role in shaping health behaviors, particularly in guiding individuals to apply nutritional knowledge to make healthy dietary choices [14]. Individuals with higher levels of nutritional literacy may be better in identifying and avoiding high-calorie, low-nutrient-density foods, thereby adopting healthier dietary patterns [15]. There are substantial variations in the determinants and risk factors of childhood obesity across age, gender, and region among Chinese school-age students [16].

Few studies have explored the scores of dietary diversities (DDS), PA and their combine impact on the risk of obesity in Chinese school-age students. Moreover, the gender-, age- and residential area-specific risk factors associated with school-age student obesity are seldom reported. Our work is scientifically meaningful for policymakers to develop evidence-based and sustainable intervention programs to control the prevalence of obesity in Chinese school-age students.

## 2. Materials and Methods

### 2.1. Study Population

Our study investigated 11,359 children aged 6–18 years in Shandong Province in 2024. A total of 74 students were excluded due to not completing the questionnaire (*n* = 2) and having a BMI > 40 (*n* = 72). In total, 11,285 participants were included in the final statistical analysis (Figure S1). This study was conducted in accordance with the Declaration of Helsinki and was approved by the Institutional Review Board (IRB) for preventive medicine at the Jining Center for disease control and prevention (No. 2023SY001). Informed consent was obtained from the students or their parents.

### 2.2. Definition of Obesity

Age- and gender-specific BMI thresholds for screening obesity in children aged 6–18 years were used for screening students with obesity. Based on “Screening for Overweight and Obesity among School-age Children and Adolescents”, the reference thresholds are shown in Table S1. In total, 1681 students were diagnosed as obese, and 9604 students were classified as non-obese.

### 2.3. Nutrition and Health Knowledge and Physical Activity Survey

The survey on nutrition health knowledge in school-age children focused on comprehensive nutritional literacy consisted of 5 parts: dietary recommendations; food safety; characteristics of food; dietary habits; and nutrition and disease. Due to the age differences, questionnaires with different content and difficulty levels for primary school grades 1–3, primary school grades 4–6, junior high school, and senior high school were conducted. The questionnaire also included behaviors of dietary frequency and physical activity questions. The original questionnaire is provided in the Supplementary Materials (Table S5).

### 2.4. DDS and PA Scoring Criteria

Based on the DDS scoring criteria [17,18] and the Chinese Dietary Guidelines [6], nine types of food intake were collected by using a self-designed questionnaire. Animal-derived foods included milk and dairy products, meat, eggs, and sea food, and plant-derived foods included cereals, whole grains, fruits, vegetables, and legumes. The Animal DDS and Plant DDS were calculated according to the score assignment criteria. Total DDS was the sum of the Animal DDS and Plant DDS (Table S2).

The Chinese Ministry of Education in China recommends that primary and secondary school students need at least 30 min of sports activities per day. Accordingly, we defined 30 min physical activity per day as 1 point, 30–59 min physical activity per day as 2 points, 60–89 min physical activity per day as 3 points, and 90 min or above per day as 4 points (Table S3). Table S4 demonstrated the grouping criteria for high and low Total DDS, Animal DDS, Plant DDS, and PA.

### 2.5. Statistical Analyses

R 4.2.3 was used for data analysis. Graphs were drafted by Graph pad Prism 8 and R 4.2.3. A Kolmogorov–Smirnov test was conducted to assess the normality of continuous variables. An independent sample *t*-test and χ^2^ test were used to compare the differences between two groups. Pearson correlation analysis was conducted to explore the association between the scores of nutritional literacy and BMI, Total DDS, Plant DDS, Animal DDS, and PA. The median method was conducted to define nutritional literacy levels for primary school, junior high school, and senior high school students. Students scoring above the median (top 50%) were classified as having high nutritional literacy, while those scoring below the median (bottom 50%) were classified as having low nutritional literacy. Logistic regression analysis was conducted, and students with low DDS, low Plant DDS, low Animal DDS, low PA, and low nutritional literacy were used as references for the whole population and in the subgroups. Confounding factors including age, gender, residential area, living at school, dining at school, household cooking responsibility, and father’s and mother’s education level were adjusted during analysis. *p* < 0.05 was considered statistical difference.

## 3. Results

### 3.1. Characteristics of the Participants

This study included 11,285 Chinese school-age students with an average age of 12.67 ± 3.46 years, including 5634 boys (49.9%) and 5651 (50.1%) girls. Significant differences were observed in age, gender, residential area, proportion of time spent living and dining at school, and parents’ education level between groups (Table 1). The total prevalence of obesity in school-age students was 14.9% (Figure S2).

Students with obesity had lower scores in Total DDS and Plant DDS but higher scores in nutritional literacy level than the control students (Table 2). Compared to the control boys, boys with obesity had lower Total DDS and lower Plant DDS but higher nutritional literacy level scores. And compared to the non-obese girls, girls with obesity had lower Plant DDS and higher nutritional literacy scores (Table 3). In the urban group, compared to the non-obese students, students with obesity had lower Plant DDS and higher PA and nutritional literacy scores. In the rural group, compared to the non-obesity group, the students with obesity had lower Total DDS and Plant DDS, with higher nutritional literacy scores (Table 4). In the primary school group, students with obesity had higher Animal DDS (Table 5).

### 3.2. Correlation of Nutritional Literacy, DDS, PA, and BMI

Positive correlations between total scores of nutritional literacy and Total DDS (r = 0.06, *p* < 0.001), Plant DDS (r = 0.07, *p* < 0.001), Animal DDS (r = 0.03, *p* < 0.01), and PA scores (r = 0.04, *p* < 0.001) and a negative correlation between total scores of nutritional literacy and BMI (r = −0.19, *p* < 0.001) were found in all the students (Figure S3). The negative correlation between total scores of nutritional literacy and BMI was found in all subgroups, except the participants in junior high school and senior high school. And we found positive correlations between total scores of nutritional literacy and Total DDS in all subgroups. In addition, positive correlations between total scores of nutritional literacy and PA were only found in girls (r = 0.11, *p* < 0.001) and students from rural areas (r = 0.06, *p* < 0.001).

### 3.3. Association of DDS and PA with the Risk of Obesity

**Figure 1:**
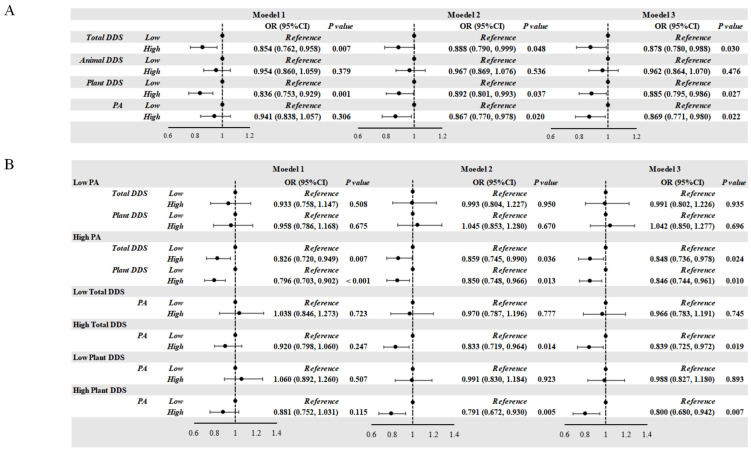

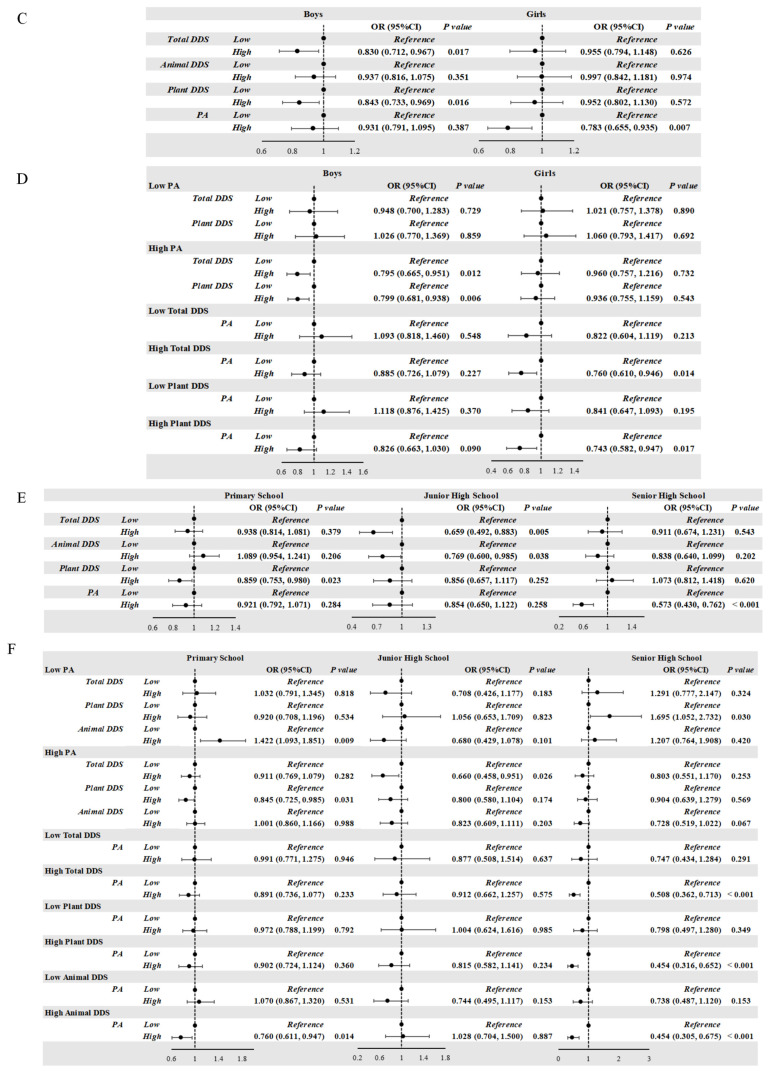

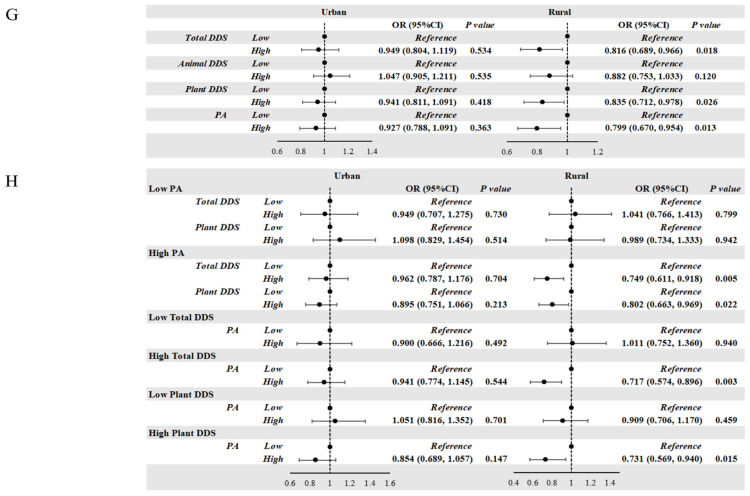
(**A**,**B**) Correlation of DDS, PA, and the risk of obesity in school-age children. (**C**,**D**) Correlation of DDS, PA, and the risk of obesity according to gender in school-age children. (**E**,**F**) Correlation of DDS, PA, and the risk of obesity according to age in school-age children. (**G**,**H**) Correlation of DDS, PA, and the risk of obesity according to residential area in school-age children. (**A**,**B**) Model 1: unadjusted; Model 2: adjusted for age and gender; Model 3: adjusted for age, gender, residential area, living at school, dining at school, household cooking responsibilities, and parents’ education level. (**C**,**D**) Model was adjusted for age, residential area, living at school, dining at school, household cooking responsibilities, and parents’ education level. (**E**,**F**) Model was adjusted for gender, residential area, living at school, dining at school, household cooking responsibilities, and parents’ education level. (**G**,**H**) Model was adjusted for age, gender, living at school, dining at school, household cooking responsibilities, and parents’ education level. DDS: dietary diversity score; PA: score of physical activity; OR: odds ratio.

Students with high Total DDS (OR = 0.878, *p* = 0.030) and high Plant DDS (OR = 0.885, *p* = 0.027) showed a lower risk of obesity (Figure 1A). After adjusting for confounding factors, the relation remained consistent. High PA was also associated with a lower risk of obesity (OR = 0.869, *p* = 0.022). We only observed high Total DDS (OR = 0.848, *p* = 0.024) and high Plant DDS (OR = 0.846, *p* = 0.010) as protective factors of obesity in the students from the high-PA group. Similarly, high PA was only observed as a protective factor for obesity in the high-Total-DDS group (OR = 0.839, *p* = 0.019) and in the high-Plant-DDS group (OR = 0.800, *p* = 0.007).

### 3.4. Association of DDS and PA with the Risk of Obesity According to Grade, Gender, and Residential Area

Compared with the boys with low Total DDS and Plant DDS levels, those with high Total DDS (OR = 0.830, *p* = 0.017) and high Plant DDS levels (OR = 0.843, *p* = 0.016) showed a significantly decreased risk of obesity. And compared with the girls with low PA levels, those with high PA levels (OR = 0.783, *p* = 0.007) displayed a decreased risk of obesity. Consistent with the results of the whole population, we only observed protective effects in the high-PA group and in the high-Total-DDS and high-Plant-DDS groups (Figure 1C–H).

Primary school students with high Plant DDS levels (OR = 0.859, *p* = 0.023), junior high school students with high Total DDS (OR = 0.659, *p* = 0.005) and Animal DDS (OR = 0.769, *p* = 0.038), and senior high school students with high PA levels (OR = 0.573, *p* < 0.001) showed a decreased risk of obesity. Among primary school students, high Plant DDS levels were associated with a decreased risk of obesity in students with high PA (OR = 0.845, *p* = 0.031). In the high-Animal-DDS group, high PA levels reduced the obesity risk (OR = 0.760, *p* = 0.014). And in the low PA group, high Animal DDS increased the obesity risk in primary school students. Among junior high school students, the protective effect of high Total DDS against obesity was observed in students with high PA levels (OR = 0.660, *p* = 0.026). Among senior high school students, high PA levels were a protective factor against obesity in students with high Total DDS (OR = 0.508, *p* < 0.001), high Plant DDS (OR = 0.454, *p* < 0.001), and high Animal DDS (OR = 0.454, *p* < 0.001). Among senior high school students with low PA levels, high Animal DDS was a risk factor for obesity when compared to low Animal DDS (OR = 1.695, *p* = 0.030).

After stratifying the students according to residential area, we did not observe significant protective effects of high Total DDS, high Plant DDS, or high PA levels on obesity among urban students, whereas these were protective factors among students in rural areas. Consistent with the findings from all students, protective effects of high Total DDS (OR = 0.749, *p* = 0.005) and high Plant DDS (OR = 0.802, *p* = 0.022) were only observed in students with high PA levels. And the protective effect of high PA was only observed in the students with high Total DDS (OR = 0.717, *p* = 0.003) and high Plant DDS levels (OR = 0.731, *p* = 0.015).

### 3.5. Association of DDS and PA with the Risk of Obesity According to the Combination of Grade, Gender, and Residential Area

High Total DDS was a protective factor against obesity among junior high school students in rural areas (OR = 0.498, *p* = 0.001), for boys (OR = 0.572, *p* = 0.034) and girls (OR = 0.377, *p* = 0.004). High PA was a protective factor against obesity among primary school girls in rural areas (OR = 0.590, *p* = 0.001), junior high school boys in rural areas (OR = 0.449, *p* = 0.004), and senior high school students in urban areas (OR = 0.561, *p* = 0.004), for boys (OR = 0.465, *p* = 0.003) and girls (OR = 0.425, *p* = 0.020) (Table 6).

### 3.6. Association of Nutritional Literacy and the Risk of Obesity

High nutritional literacy was a risk factor for obesity only among rural primary school students (OR = 1.365, *p* = 0.002), and this association was especially significant in girls (OR = 2.167, *p* < 0.001) (Table 7 and Table 8).

## 4. Discussion

This study revealed significant gender-specific differences in the prevalence of obesity, with 18.7% of boys and 11.1% of girls being obese, a pattern consistent with conditions in some countries. A study involving 16 European countries found that the obesity rate among males (14.0%) was higher than that among females (11.5%). The obesity rate among women in the United States (40%) is higher than that among men (35%); in Italy, while the prevalence of overweight in males (40.2%) is significantly higher than in females (27.4%), the prevalence of obesity in females (9.6%) is lower than in males (17.6%). This suggests that the distribution of obesity prevalence between genders varies across countries, shaped by a range of factors including biology, behavior, and socio-economic conditions [2]. Biologically, females have lower calorie needs and higher body fat content and leptin levels [19], which may contribute to the gender difference in obesity prevalence [20]. Social and cultural factors also contribute to the gender difference in obesity prevalence. Socially, cultural expectations of females being “slim” lead parents to control girls’ body weight during upbringing. A survey conducted on Chinese adolescents showed that 41.6% of girls with a normal body weight or who are underweight perceive themselves as overweight compared to 11.6% of boys [21]. Girls are more weight-conscious and adjust their diet and lifestyle [22,23]. In addition, parents of children without obesity tend to have high education levels, which is another potential influencing factor on childhood obesity [24]. Moreover, girls have lower food intake and total DDS scores compared to the boys, demonstrating rigorous quantity control of diet intake.

The highest incidence of overweight and obesity among adolescents were in North and Northeast China [25]. Data from National surveys showed that, in 2019, urban children had a higher prevalence of obesity than rural children (13.4% for urban boys and 7.3% for urban girls; 10.9% for rural boys and 6.6% for rural girls) [26]. Consistently, we observed the region-dependent prevalence of obesity in students (15.8% and 13.9% in urban and rural areas). Uneven economic development between urban and rural areas leads to lifestyle and food access differences. Urban children have abundant energy-dense food supplies, while rural diets mainly feature local grains, vegetables, and livestock. Despite recent changes in rural food access, urban areas still have an advantage in food supply diversity and convenience [27]. Our comparison of urban–rural differences in obesity is consistent with the association between urbanization and obesity rates seen globally. Across the world, as urbanization advances, obesity rates are on the rise. Urban areas have greater access to processed foods and sugary drinks, and the mechanization of transportation has reduced physical activity, both of which contribute to higher obesity rates. Taking Chile as an example, it is notable that although the urban–rural difference in obesity rates is not distinct, shifts in dietary patterns, such as a preference for high-fat, high-sugar, and high-salt foods, have led to an increase in overweight and obesity, and this trend is likely more pronounced in urban areas [2].

The prevalence of obesity varies by grade among school-age students, with 20.4% in primary school, 10.1% in junior high school, and 8.8% in senior high school. Students with obesity across all grades have low vegetable, seafood, meat, and whole grain intake. However, junior high school students show greater food diversity, suggesting a shift in food choices. School-age children need diverse foods and sufficient nutrients for normal growth [28]. Vegetables, meat, and seafood are rich in various nutrients essential for bone, muscle, and immune system development [29,30,31]. Low intake of vegetables, meat, and seafood can lead to nutritional imbalance and abnormal energy metabolism, increasing obesity risk. In junior high school, with continued physical development and puberty, the demand for energy and nutrients diversifies correspondingly [32]. Whole grains are rich in dietary fiber and B vitamins [33]. Insufficient whole grain intake may disrupt blood glucose levels, affecting satiety and weight control [34]. In senior high school, while physical development nears maturity, academic pressure peaks [35]. Students have less time for outdoor activities and choose sedentary leisure with electronic devices [36,37]. Senior high school students with obesity consume meat most frequently. Meat is a good source of protein and iron; however, the over-consumption of red meat raises the risk of obesity and cardiovascular diseases [38]. Thus, students should consume animal-derived foods reasonably.

Children with obesity displayed higher nutritional literacy than healthy students. From the children’s perspective, although they clearly knew the importance of reasonable nutrition, factors such as dietary preference, food temptations, and poor self-control prevented them from applying their nutritional knowledge [39]. Unhealthy parental eating habits limit the availability of whole grains and plant-based foods and increase fast-food consumption, influencing children’s dietary habits [40]. School nutritional education is helpful for literacy but has a limited impact on changing students’ dietary habits [41]. School canteens lack diverse healthy options or effective diet monitoring [42]. In addition, the widespread availability of high-calorie, processed foods and their advertisements pose strong temptations for children with obesity, overwhelming their limited nutritional knowledge [43]. Children with obesity may experience emotional distress, which can trigger emotional eating, creating a vicious cycle [44]. Some children also have wrong ideas about weight loss, focusing only on reducing intake [45]. Children with obesity and their parents often receive more health education due to the child’s obesity. However, the physiological changes associated with puberty and the time required for behavioral adjustments mean that they may not immediately transition out of an obese state.

High-level Total DDS, plant-based DDS, and PA are commonly suggested protective factors for the prevention of obesity, but they vary by gender, educational stage, and region. For school-age children, high levels of daily PA and DDS protect against childhood obesity. Children who perform over 30 min of exercise daily and who have a weekly Total DDS score above 12 are at a low risk for obesity. The relation between BMI, DDS, nutritional literacy, and PA are complex, showing an intricate interaction between physical development, metabolic traits, and lifestyle factors. Dietary diversity and balanced diets support energy metabolism and tissue repair during PA. Adequate PA boosts energy expenditure, balancing energy intake and consumption to maintain a healthy weight. Processed foods and minimal PA lead to excess energy turning into fat. Plant-based diverse diets that are rich in fiber regulate gut microbiota, energy, and lipid metabolism. Regular PA increases muscle insulin sensitivity and glucose utilization and reduces lipid synthesis.

Our findings on childhood obesity are closely aligned with national policies, offering actionable insights to formulate targeted public health and education strategies. Firstly, the policy of elevating per capita subsidies for basic public health services is designed to ameliorate the populace’s health status while concurrently furnishing a pivotal fiscal framework for nutritional intervention endeavors [46]. Our research underscores the finding that rural students with obesity exhibit inferior dietary diversity, and subsidizing farmers to expand the cultivation of fruits, vegetables, and grains constitutes a viable strategy to bridge this disparity. Such an approach, by enhancing dietary diversity, contributes to the equilibrium of regional health development and mitigates the risk of obesity. Secondly, national initiatives encouraging population-wide exercise, particularly among children, resonate with our findings that targeted activity reduces obesity risk, especially for rural junior high school boys and urban senior high school students. Enhancing public health awareness supports the refinement of policies aimed at funding rural sports facilities and tailored urban extracurricular activities, and this approach advances the policy’s goal of fostering healthy lifestyles through strategic, group-specific interventions [47]. Finally, nutritional knowledge needs vary by school level—foundational habits for primary students, dietary health links for juniors, and long-term health impacts for seniors. Adapting curricula to these needs, coupled with teacher training, directly supports the ministry’s mandate for relevant, effective health education [48].

The strengths of this study lie in its focus on Chinese school-age children, with stratification by age, gender, and region to explore the associations between nutrition and health knowledge, dietary diversity, physical activity, and obesity, providing a basis for personalized obesity prevention. Additionally, our study employs a large sample size (11,285 students aged 6–18 years) and multivariate analysis (adjusting for confounding factors such as age and gender), enhancing the reliability of the results. This study also has several limitations. Firstly, the participants were mainly recruited from Shandong Province. Due to regional and food cultural differences across different regions of China, the extrapolation of our conclusions should be undertaken with caution. Secondly, this cross-sectional study cannot explain the in-depth interactions and underlying mechanisms of nutritional literacy and physical activity in affecting obesity risk. Lastly, given that the data pertaining to our research subjects was self-reported, recall bias is unavoidable. This underscores the need for prospective studies to address such limitations in future research.

## 5. Conclusions

A combination of diverse dietary intakes and engaging in more than 30 min of daily physical activity is essential for reducing the risk of obesity among Chinese school-age students. Notably, to prevent obesity, rural junior high school students need to increase dietary diversity, and primary school girls in rural areas, junior high school boys in rural areas, and senior high school students in urban areas need to enhance their daily outdoor physical activity. In future research, cohort studies should be conducted to investigate the exact causal relationships between nutrition literacy, dietary diversity, physical activity, and the occurrence of obesity. Such studies could provide more robust evidence to guide interventions among school-age students.

## Figures and Tables

**Table 1:** Demographic characteristics of the school-age students (*n* = 11,285).

Demographic Characteristics	Obesity (*n* = 1681)	Control (*n* = 9604)	Total (*n* = 11,285)	*p* Value
Age (years)	11.32 ± 3.28	12.91 ± 3.44	12.67 ± 3.46	<0.001
BMI (kg/m^2^)	27.16 ± 4.67	18.65 ± 3.25	19.92 ± 4.63	<0.001
Gender, *n* (%)				<0.001
Boys	1052 (62.6)	4582 (47.7)	5634 (49.9)	
Girls	629 (37.4)	5022 (52.3)	5651 (50.1)	
Residential area, *n* (%)				0.005
Urban	932 (55.4)	4965 (51.7)	5897 (52.3)	
Rural	749 (44.6)	4639 (48.3)	5388 (47.7)	
Living at school, *n* (%)				<0.001
Yes	217 (12.9)	2281 (23.8)	2498 (22.1)	
No	1464 (87.1)	7323 (76.2)	8787 (77.9)	
Dining at school, *n* (%)				<0.001
Yes	801 (47.7)	5895 (61.4)	6696 (59.3)	
No	880 (52.3)	3709 (38.6)	4589 (40.7)	
Cooking responsibility, *n* (%)				0.189
Father	190 (11.3)	1123 (11.7)	1313 (11.6)	
Mother	1030 (61.3)	6073 (63.2)	7103 (62.9)	
Grandparents	419 (24.9)	2214 (23.1)	2633 (23.3)	
Others	42 (2.5)	194 (2.0)	236 (2.1)	
Father’s education, *n* (%)				<0.001
Primary school	115 (6.8)	489 (5.1)	604 (5.4)	
Junior high school	646 (38.4)	4296 (44.7)	4942 (43.8)	
High school	461 (27.4)	2590 (27.0)	3051 (27.0)	
Junior college and above	309 (18.4)	1566 (16.3)	1875 (16.6)	
Unknown	150 (8.9)	663 (6.9)	813 (7.2)	
Mother’s education, *n* (%)				<0.001
Primary school	220 (13.1)	1163 (12.1)	1383 (12.3)	
Junior high school	650 (38.7)	4278 (44.5)	4928 (43.7)	
High school	355 (21.1)	2035 (21.2)	2390 (21.2)	
Junior college and above	284 (16.9)	1458 (15.2)	1742 (15.4)	
Unknown	172 (10.2)	670 (7.0)	842 (7.5)	

Data is presented as mean ± standard or *n* (%). *T*-test was used to compare age and BMI; χ^2^ test was used to compare gender, residential area, living and dining at school, cooking responsibility, and parents’ education level. *p* < 0.05 was considered statistically significant.

**Table 2:** Dietary intake and physical exercise of the school-age students.

Variables	Obesity (*n* = 1681)	Control (*n* = 9604)	Total (*n* = 11,285)	*p* Value
Dietary Intake (Times/Week)				
Cereals	5.436 ± 2.262	5.627 ± 2.121	5.598 ± 2.144	0.001
Vegetables	5.430 ± 2.182	5.627 ± 2.020	5.598 ± 2.046	0.001
Fruits	5.124 ± 2.254	5.210 ± 2.172	5.197 ± 2.184	0.150
Dairy	4.805 ± 2.389	4.813 ± 2.334	4.812 ± 2.342	0.906
Legumes	3.899 ± 2.496	3.973 ± 2.463	3.962 ± 2.468	0.256
Eggs	4.891 ± 2.401	4.940 ± 2.342	4.933 ± 2.351	0.434
Meat	4.639 ± 2.257	4.679 ± 2.212	4.673 ± 2.219	0.492
Whole grain	4.540 ± 2.411	4.554 ± 2.325	4.552 ± 2.337	0.825
Sea foods	2.792 ± 2.496	2.716 ± 2.393	2.728 ± 2.409	0.247
DDS (points)				
Total DDS	13.256 ± 3.190	13.438 ± 3.187	13.411 ± 3.188	0.032
Animal DDS	5.569 ± 1.711	5.588 ± 1.666	5.585 ± 1.673	0.666
Plant DDS	7.688 ± 1.895	7.850 ± 1.886	7.826 ± 1.888	0.001
PA (points)	2.233 ± 1.005	2.251 ± 1.036	2.236 ± 1.010	0.514
Total nutritional literacy (points)	77.53 ± 16.71	74.75 ± 15.59	75.17 ± 15.79	<0.001

DDS, scores of dietary diversity; PA, physical activity.

**Table 3:** Dietary intake and physical exercise of the school-age students according to gender.

Variables	Obesity		Control		*p* Value
	Boys (*n* = 1052)	Girls (*n* = 629)	Boys (*n* = 4582)	Girls (*n* = 5022)	
Dietary intakes (times/week)					
Cereals	5.554 ± 2.200	5.240 ± 2.351 ^a^	5.777 ± 2.044 ^b^	5.490 ± 2.181 ^a,b^	<0.001
Vegetables	5.435 ± 2.182	5.422 ± 2.184	5.619 ± 2.017 ^b^	5.635 ± 2.022 ^b^	0.004
Fruits	5.130 ± 2.244	5.114 ± 2.271	5.182 ± 2.200	5.234 ± 2.146	0.313
Dairy	4.890 ± 2.374	4.664 ± 2.409	4.987 ± 2.320	4.654 ± 2.336 ^a^	<0.001
Legumes	4.021 ± 2.491	3.694 ± 2.493 ^a^	4.180 ± 2.458	3.784 ± 2.453 ^a^	<0.001
Eggs	4.940 ± 2.377	4.809 ± 2.441	5.099 ± 2.294 ^b^	4.795 ± 2.377 ^a^	<0.001
Meat	4.939 ± 2.185	4.137 ± 2.288 ^a^	4.934 ± 2.149	4.446 ± 2.243 ^a,b^	<0.001
Whole grain	4.594 ± 2.412	4.451 ± 2.407	4.649 ± 2.320	4.468 ± 2.326 ^a^	0.001
Sea foods	2.919 ± 2.564	2.580 ± 2.363 ^a^	2.867 ± 2.471	2.579 ± 2.312 ^a^	<0.001
DDS (points)					
Total DDS	13.446 ± 3.208	12.940 ± 3.135 ^a^	13.703 ± 3.172 ^b^	13.195 ± 3.182 ^a^	<0.001
Animal DDS	5.696 ± 1.685	5.356 ± 1.734 ^a^	5.756 ± 1.643	5.434 ± 1.673 ^a^	<0.001
Plant DDS	7.750 ± 1.921	7.583 ± 1.846	7.947 ± 1.887 ^b^	7.761 ± 1.882 ^a,b^	<0.001
PA (points)	2.384 ± 1.051	2.029 ± 0.972 ^a^	2.432 ± 1.047	2.052 ± 0.929 ^a^	<0.001
Total nutritional literacy (points)	76.04 ± 17.15	80.04 ± 15.63 ^a^	74.10 ± 16.40 ^b^	75.34 ± 14.78 ^a,b^	<0.001

DDS, scores of dietary diversity; PA, physical activity. a: vs. boys; b: vs. obesity.

**Table 4:** Dietary intake and physical exercise of the school-age students according to residential area.

Variables	Obesity		Control		*p* Value
	Urban (*n* = 932)	Rural (*n* = 749)	Urban (*n* = 4965)	Rural (*n* = 4639)	
Dietary intakes (times/week)					
Cereals	5.378 ± 2.276	5.509 ± 2.244	5.563 ± 2.149 ^a^	5.695 ± 2.090 ^a,b^	<0.001
Vegetables	5.519 ± 2.177	5.320 ± 2.185 ^b^	5.725 ± 1.942 ^a^	5.523 ± 2.095 ^a,b^	<0.001
Fruits	5.343 ± 2.192	4.852 ± 2.301 ^b^	5.306 ± 2.098	5.107 ± 2.244 ^a,b^	<0.001
Dairy	4.831 ± 2.430	4.774 ± 2.339	4.939 ± 2.291	4.678 ± 2.372 ^b^	<0.001
Legumes	3.951 ± 2.506	3.833 ± 2.483	4.023 ± 2.438	3.919 ± 2.489	0.088
Eggs	4.939 ± 2.395	4.831 ± 2.410	4.905 ± 2.323	4.978 ± 2.363	0.278
Meat	4.761 ± 2.236	4.486 ± 2.275 ^b^	4.814 ± 2.172	4.535 ± 2.245 ^b^	<0.001
Whole grain	4.620 ± 2.387	4.441 ± 2.437	4.585 ± 2.308	4.521 ± 2.342	0.230
Sea foods	2.948 ± 2.554	2.598 ± 2.409 ^b^	2.752 ± 2.365 ^a^	2.678 ± 2.423	0.006
DDS (points)					
Total DDS	13.437 ± 3.168	13.032 ± 3.204 ^b^	13.578 ± 3.057	13.288 ± 3.314 ^a,b^	<0.001
Animal DDS	5.652 ± 1.724	5.465 ± 1.690 ^b^	5.661 ± 1.615	5.510 ± 1.716 ^b^	<0.001
Plant DDS	7.784 ± 1.891	7.567 ± 1.894 ^b^	7.917 ± 1.817 ^a^	7.778 ± 1.955 ^a,b^	<0.001
PA (points)	2.255 ± 1.036	2.246 ± 1.037	2.180 ± 0.992 ^a^	2.290 ± 1.015 ^b^	<0.001
Total nutritional literacy (points)	77.93 ± 15.71	77.04 ± 17.87	75.44 ± 14.67 ^a^	74.01 ± 16.48 ^a,b^	<0.001

DDS, scores of dietary diversity; PA, physical activity. a: vs. obesity; b: vs. urban.

**Table 5:** Dietary intake and physical exercise of the school-age students according to grade.

Variables	Obesity			Control			*p* Value
	Primary School (*n* = 1141)	Junior High School (*n* = 303)	Senior High School (*n* = 237)	Primary (*n* = 4441)	Junior High School (*n* = 2700)	Senior High School (*n* = 2463)	
Dietary intake (times/week)							
Cereals	5.046 ± 2.418	6.353 ± 1.476 ^a^	6.146 ± 1.755 ^a^	5.022 ± 2.367	6.314 ± 1.561 ^a^	5.963 ± 1.868 ^a,b^	<0.001
Vegetables	5.218 ± 2.301	6.041 ± 1.731 ^a^	5.669 ± 1.932 ^a,b^	5.368 ± 2.170 ^c^	6.027 ± 1.726 ^a^	5.657 ± 1.963 ^a,b^	<0.001
Fruits	5.144 ± 2.274	5.337 ± 2.100	4.755 ± 2.313 ^a,b^	5.227 ± 2.171	5.458 ± 2.028 ^a^	4.906 ± 2.289 ^a,b^	<0.001
Dairy	4.770 ± 2.431	5.117 ± 2.250 ^a^	4.580 ± 2.330 ^b^	4.619 ± 2.408	5.221 ± 2.177 ^a^	4.714 ± 2.310 ^b^	<0.001
Legumes	3.699 ± 2.542	4.554 ± 2.262 ^a^	4.023 ± 2.413 ^b^	3.613 ± 2.477	4.646 ± 2.322 ^a^	3.885 ± 2.438 ^a,b^	<0.001
Eggs	4.845 ± 2.457	4.983 ± 2.302	4.994 ± 2.252	4.808 ± 2.413	5.120 ± 2.245 ^a^	4.982 ± 2.304 ^a,b^	<0.001
Meat	4.360 ± 2.310	5.182 ± 2.067 ^a^	5.285 ± 1.963 ^a^	4.176 ± 2.263 ^c^	5.230 ± 2.029 ^a^	4.981 ± 2.110 ^a,b,c^	<0.001
Whole grain	4.555 ± 2.418	4.508 ± 2.384	4.513 ± 2.418	4.527 ± 2.301	4.909 ± 2.258 ^a,c^	4.216 ± 2.385 ^a,b^	<0.001
Sea foods	2.780 ± 2.472	3.030 ± 2.526	2.546 ± 2.556 ^b^	2.581 ± 2.307 ^c^	3.101 ± 2.463 ^a^	2.538 ± 2.421 ^b^	<0.001
DDS (points)							
Total DDS	12.972 ± 3.155	14.172 ± 3.153 ^a^	13.456 ± 3.176 ^a,b^	12.941 ± 3.093	14.344 ± 3.033 ^a^	13.339 ± 3.304 ^a,b^	<0.001
Animal DDS	5.474 ± 1.714	5.898 ± 1.715 ^a^	5.603 ± 1.643 ^b^	5.364 ± 1.653 ^c^	5.954 ± 1.626 ^a^	5.589 ± 1.663 ^a,b^	<0.001
Plant DDS	7.498 ± 1.909	8.274 ± 1.711 ^a^	7.852 ± 1.889 ^a,b^	7.577 ± 1.882	8.390 ± 1.707 ^a^	7.750 ± 1.958 ^a,b^	<0.001
PA (points)	2.319 ± 1.046	2.165 ± 0.986 ^a^	2.034 ± 1.016 ^a^	2.316 ± 1.032	2.177 ± 0.974 ^a^	2.146 ± 0.977 ^a^	<0.001
Total nutritional literacy (points)	82.25 ± 15.51	70.25 ± 14.76 ^a^	64.14 ± 13.81 ^a,b^	82.07 ± 14.30	71.00 ± 14.01 ^a^	65.66 ± 13.00 ^a,b^	<0.001

Data is presented as mean ± standard or *n* (%). Analysis of variance was used to compare the frequency of dietary intake, DDS, scores of PA, and total nutritional literacy. DDS, scores of dietary diversity; PA, physical activity. a: vs. primary school; b: vs. junior high school; c: vs. obesity.

**Table 6:** Association of Total DDS (12–18 vs. 0–11), Plant DDS (8–10 vs. 0–7), and PA (2–4 vs. 1) in school-age students with the risk of obesity according to gender, grade, and residential area.

Grade	Region	Gender	*n*	OR 95%CI	*p*	OR 95%CI	*p*	OR 95%CI	*p*
				High Total DDS (12–18)		High Plant DDS (8–10)		High PA (2–4)	
Primary School	Urban	Total	2876	0.972 (0.802, 1.178)	0.770	0.876 (0.743, 1.045)	0.141	1.005 (0.820, 1.233)	0.959
		Boys	1458	0.808 (0.626, 1.044)	0.103	0.810 (0.640, 1.026)	0.080	0.917 (0.693, 1.212)	0.542
		Girls	1418	1.167 (0.865, 1.574)	0.311	0.950 (0.723, 1.248)	0.712	1.027 (0.757, 1.392)	0.864
	Rural	Total	2706	0.932 (0.757, 1.147)	0.507	0.863 (0.710, 1.049)	0.138	0.911 (0.730, 1.136)	0.409
		Boys	1357	0.969 (0.733, 1.281)	0.825	0.788 (0.609, 1.021)	0.072	1.240 (0.895, 1.718)	0.196
		Girls	1349	0.854 (0.623, 1.171)	0.327	0.923 (0.681, 1.250)	0.605	0.590 (0.430, 0.809)	0.001
Junior High School	Urban	Total	1686	1.001 (0.648, 1.547)	0.996	1.112 (0.769, 1.610)	0.572	1.328 (0.934, 1.887)	0.114
		Boys	839	0.839 (0.478, 1.474)	0.542	1.145 (0.707, 1.854)	0.583	1.500 (0.906, 2.482)	0.115
		Girls	847	1.063 (0.516, 2.189)	0.868	0.881 (0.482, 1.611)	0.682	0.746 (0.428, 1.302)	0.302
	Rural	Total	1317	0.498 (0.334, 0.743)	0.001	0.703 (0.479, 1.031)	0.071	0.669 (0.445, 1.006)	0.054
		Boys	643	0.572 (0.342, 0.958)	0.034	0.750 (0.464, 1.213)	0.241	0.449 (0.263, 0.769)	0.004
		Girls	674	0.377 (0.192, 0.737)	0.004	0.652 (0.336, 1.268)	0.208	0.740 (0.375, 1.460)	0.385
Senior High School	Urban	Total	1335	1.116 (0.703, 1.771)	0.640	1.356 (0.893, 2.061)	0.153	0.561 (0.376, 0.836)	0.004
		Boys	661	0.830 (0.475, 1.452)	0.514	1.113 (0.670, 1.850)	0.680	0.465 (0.278, 0.776)	0.003
		Girls	674	1.507 (0.635, 3.574)	0.352	1.646 (0.767, 3.529)	0.201	0.425 (0.207, 0.874)	0.020
	Rural	Total	1365	0.894 (0.604, 1.324)	0.557	0.983 (0.678, 1.426)	0.929	0.802 (0.540, 1.191)	0.275
		Boys	676	0.822 (0.493, 1.373)	0.454	0.905 (0.563, 1.455)	0.681	0.600 (0.357, 1.009)	0.054
		Girls	689	0.849 (0.445, 1.620)	0.620	1.079 (0.576, 2.023)	0.813	0.852 (0.447, 1.625)	0.627

Model was adjusted for living at school, dining at school, household cooking responsibilities, and parents’ education level. DDS, scores of dietary diversity; PA, physical activity.

**Table 7:** Association of nutritional literacy with the risk of obesity in all students and according to grade.

Group	*n*	OR 95%CI	*p*
All	11,285	1.087 (0.979, 1.207)	0.117
Primary School (85–100 points vs. 0–84 points)	5582	1.125 (0.986, 1.282)	0.079
Junior High School (75–100 points vs. 0–74 points)	3003	1.021 (0.804, 1.297)	0.866
Senior High School (68–100 points vs. 0–67 points)	2700	0.886 (0.676, 1.160)	0.378

**Table 8:** Association of nutritional literacy with the risk of obesity according to gender, grade, and residential area in school-age students.

Grade	Region	Gender	*n*	OR 95%CI	*p*
Primary School (85–100 points vs. 0–84 points)	Urban	Total	2876	0.953 (0.798, 1.139)	0.599
		Boys	1458	1.028 (0.811, 1.302)	0.819
		Girls	1418	0.887 (0.673, 1.169)	0.394
	Rural	Total	2706	1.365 (1.121, 1.662)	0.002
		Boys	1357	1.026 (0.793, 1.328)	0.845
		Girls	1349	2.167 (1.571, 2.989)	<0.001
Junior High School (75–100 points vs. 0–74 points)	Urban	Total	1686	1.097 (0.804, 1.499)	0.559
		Boys	839	1.271 (0.865, 1.866)	0.222
		Girls	847	0.835 (0.477, 1.461)	0.528
	Rural	Total	1317	0.898 (0.614, 1.313)	0.580
		Boys	643	0.846 (0.525, 1.361)	0.489
		Girls	674	1.012 (0.525, 1.950)	0.971
Senior High School (68–100 points vs. 0–67 points)	Urban	Total	1335	0.801 (0.541, 1.185)	0.267
		Boys	661	0.722 (0.448, 1.164)	0.181
		Girls	674	0.995 (0.486, 2.038)	0.990
	Rural	Total	1365	0.984 (0.677, 1.429)	0.932
		Boys	676	1.091 (0.676, 1.758)	0.722
		Girls	689	0.992 (0.531, 1.854)	0.981

Model was adjusted for living at school, dining at school, household cooking responsibilities, and parents’ education level.

## Data Availability

The datasets generated during and analyzed during the current study are available from the corresponding author on reasonable request.

## Supplementary Materials

The following supporting information can be downloaded at: https://www.mdpi.com/article/10.3390/nu17132214/s1, Figure S1: Flow chart of the study participants selection process; Figure S2: Prevalence of obesity among school-age students according to gender, grade, and residential areas; Figure S3: Correlation of Total scores of Nutritional Literacy with BMI, Total DDS, Plant DDS, Animal DDS, PA in the school-age children; Table S1: Diagnostic criteria of child obesity (kg/m^2^); Table S2: Dietary frequency grading and scoring; Table S3: Physical activity grading and scoring; Table S4: DDS and PA Score Grading; Table S5: Partial questionnaire presentation.

## Abbreviations

The following abbreviations are used in this manuscript:

PA	Physical Activity
DDS	Dietary Diversity Scores
WHO	World Health Organization
AHA	American Heart Association
BMI	Body Mass Index
