# Supplementary material for: Gender-, Age-, and Region-Specific Associations Between Obesity and Nutrition/Health Knowledge, Dietary Diversity, and Physical Activity in Chinese School-Age Students: A Cross-Sectional Study
Source: Nutrients. 2025 Jul 3;17(13):2214. doi: 10.3390/nu17132214 (PMC12252032; doi:10.3390/nu17132214)
Supplement: Supplementary file 1 [file nutrients-17-02214-s001.zip › Supplementary Figure.pptx]

## Slide 1
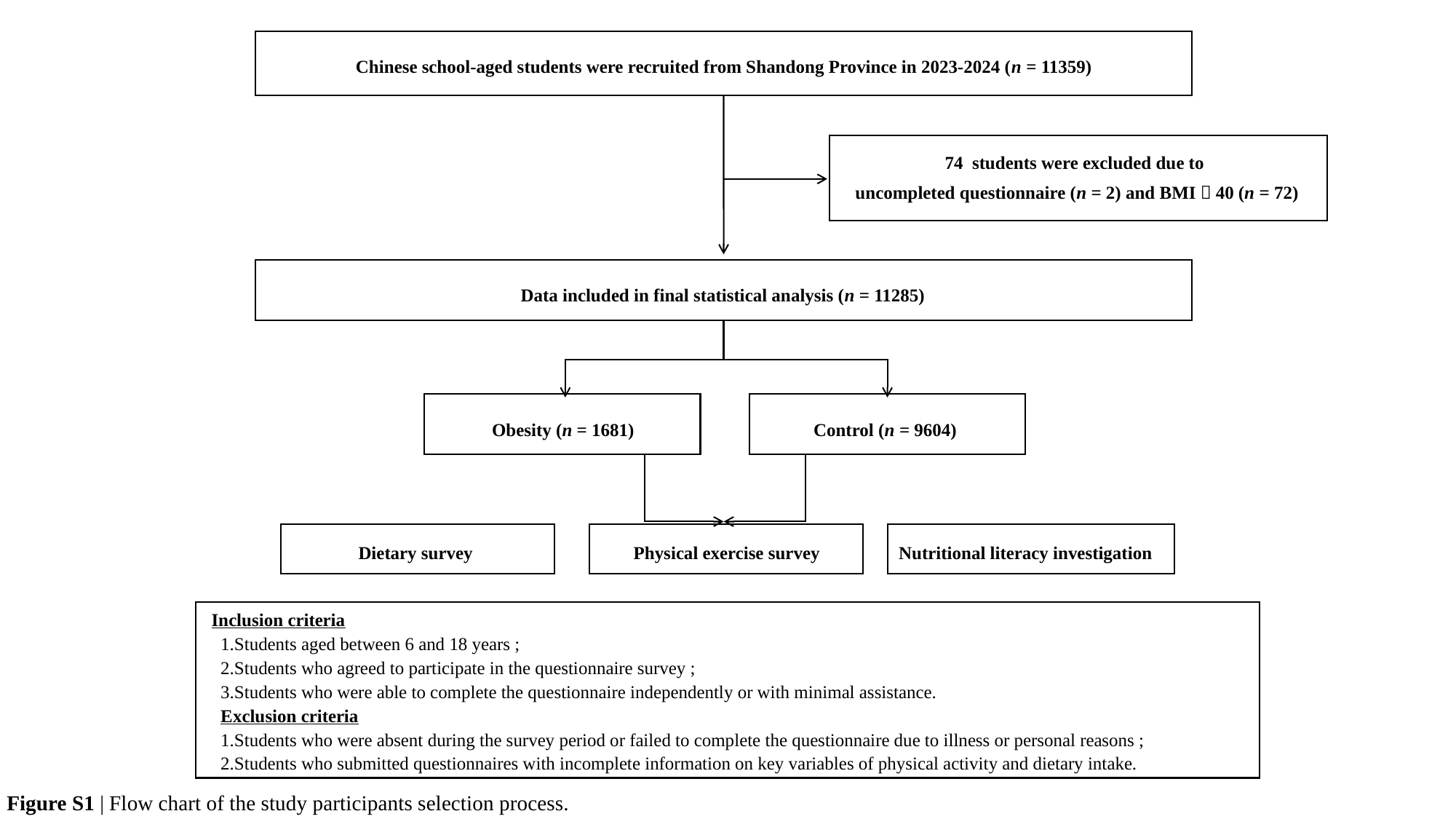

Chinese school-aged students were recruited from Shandong Province in 2023-2024 (n = 11359)
74 students were excluded due to
uncompleted questionnaire (n = 2) and BMI＞40 (n = 72)
Data included in final statistical analysis (n = 11285)
Obesity (n = 1681)
Control (n = 9604)
Dietary survey
Physical exercise survey
Nutritional literacy investigation
Inclusion criteria
1.Students aged between 6 and 18 years ;
2.Students who agreed to participate in the questionnaire survey ;
3.Students who were able to complete the questionnaire independently or with minimal assistance.
Exclusion criteria
1.Students who were absent during the survey period or failed to complete the questionnaire due to illness or personal reasons ;
2.Students who submitted questionnaires with incomplete information on key variables of physical activity and dietary intake.
Figure S1 | Flow chart of the study participants selection process.

## Slide 2
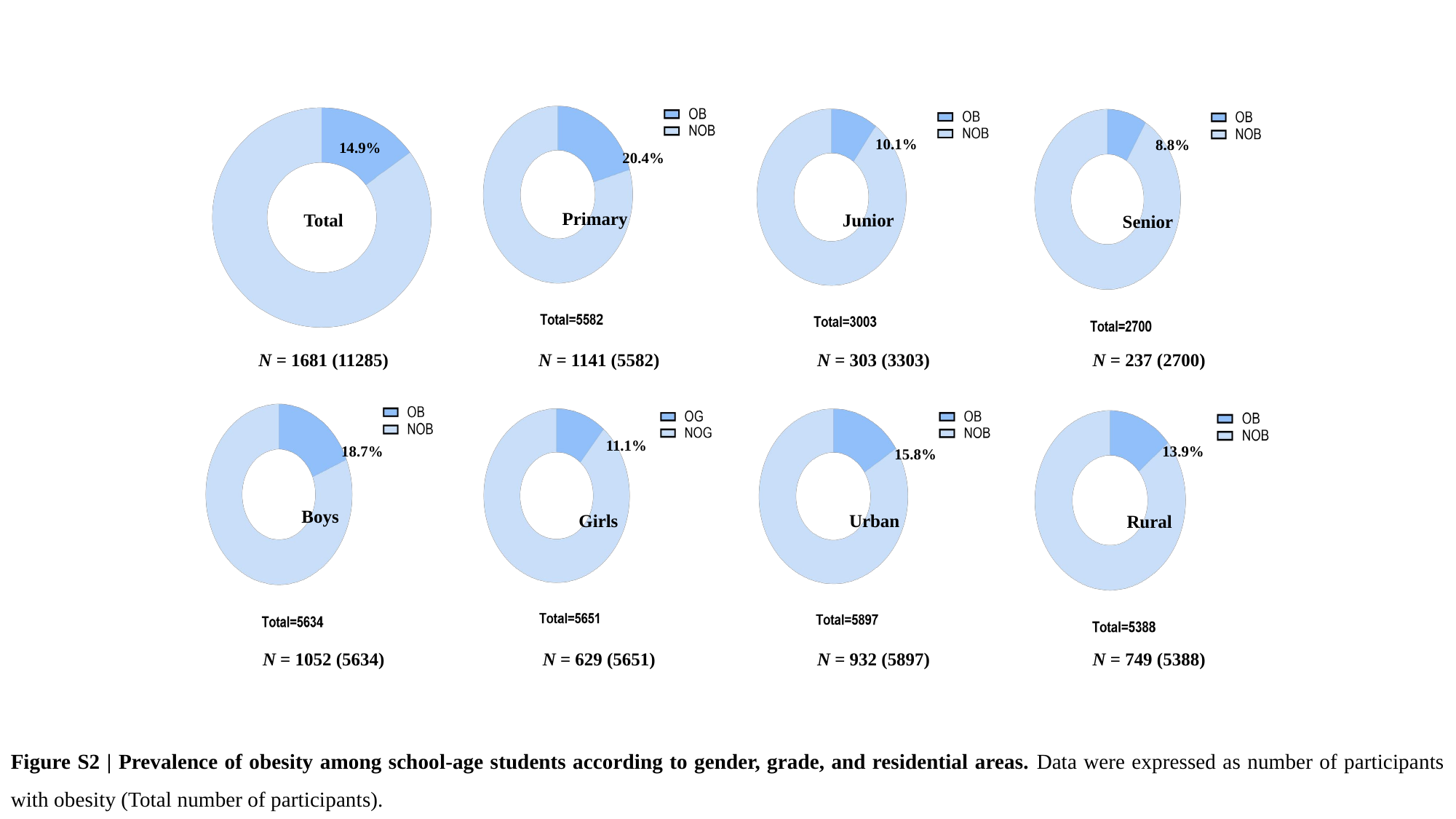

20.4%
Primary
10.1%
Junior
8.8%
Senior
14.9%
Total
N = 1681 (11285)
N = 1141 (5582)
N = 303 (3303)
N = 237 (2700)
18.7%
Boys
11.1%
Girls
15.8%
Urban
13.9%
Rural
N = 1052 (5634)
N = 629 (5651)
N = 932 (5897)
N = 749 (5388)
Figure S2 | Prevalence of obesity among school-age students according to gender, grade, and residential areas. Data were expressed as number of participants with obesity (Total number of participants).

## Slide 3
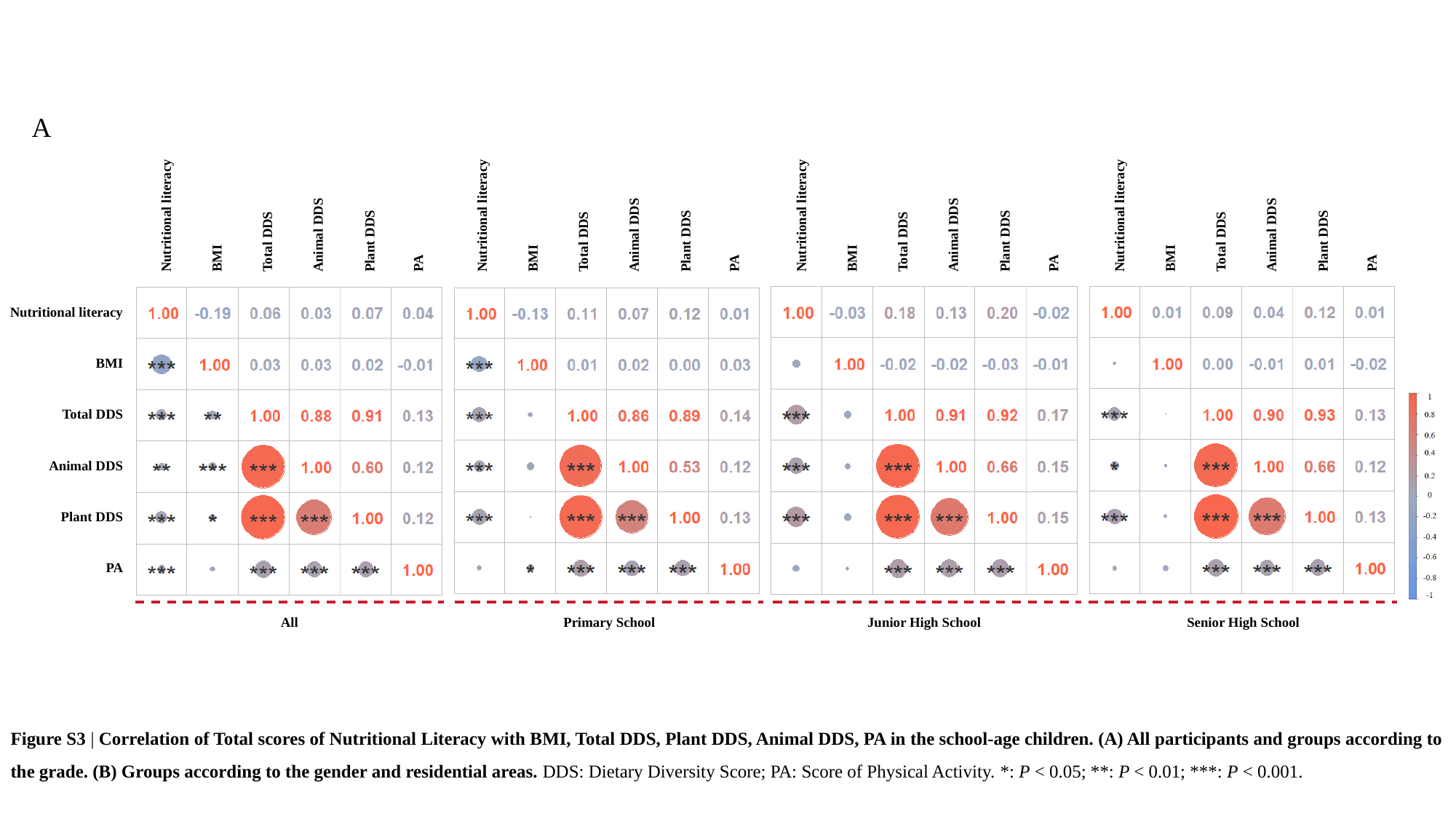

A
Nutritional literacy
BMI
Total DDS
Animal DDS
Plant DDS
PA
Nutritional literacy
BMI
Total DDS
Animal DDS
Plant DDS
PA
Nutritional literacy
BMI
Total DDS
Animal DDS
Plant DDS
PA
Nutritional literacy
BMI
Total DDS
Animal DDS
Plant DDS
PA
Nutritional literacy
BMI
Total DDS
Animal DDS
Plant DDS
PA
All
Primary School
Junior High School
Senior High School
Figure S3 | Correlation of Total scores of Nutritional Literacy with BMI, Total DDS, Plant DDS, Animal DDS, PA in the school-age children. (A) All participants and groups according to the grade. (B) Groups according to the gender and residential areas. DDS: Dietary Diversity Score; PA: Score of Physical Activity. *: P < 0.05; **: P < 0.01; ***: P < 0.001.

## Slide 4
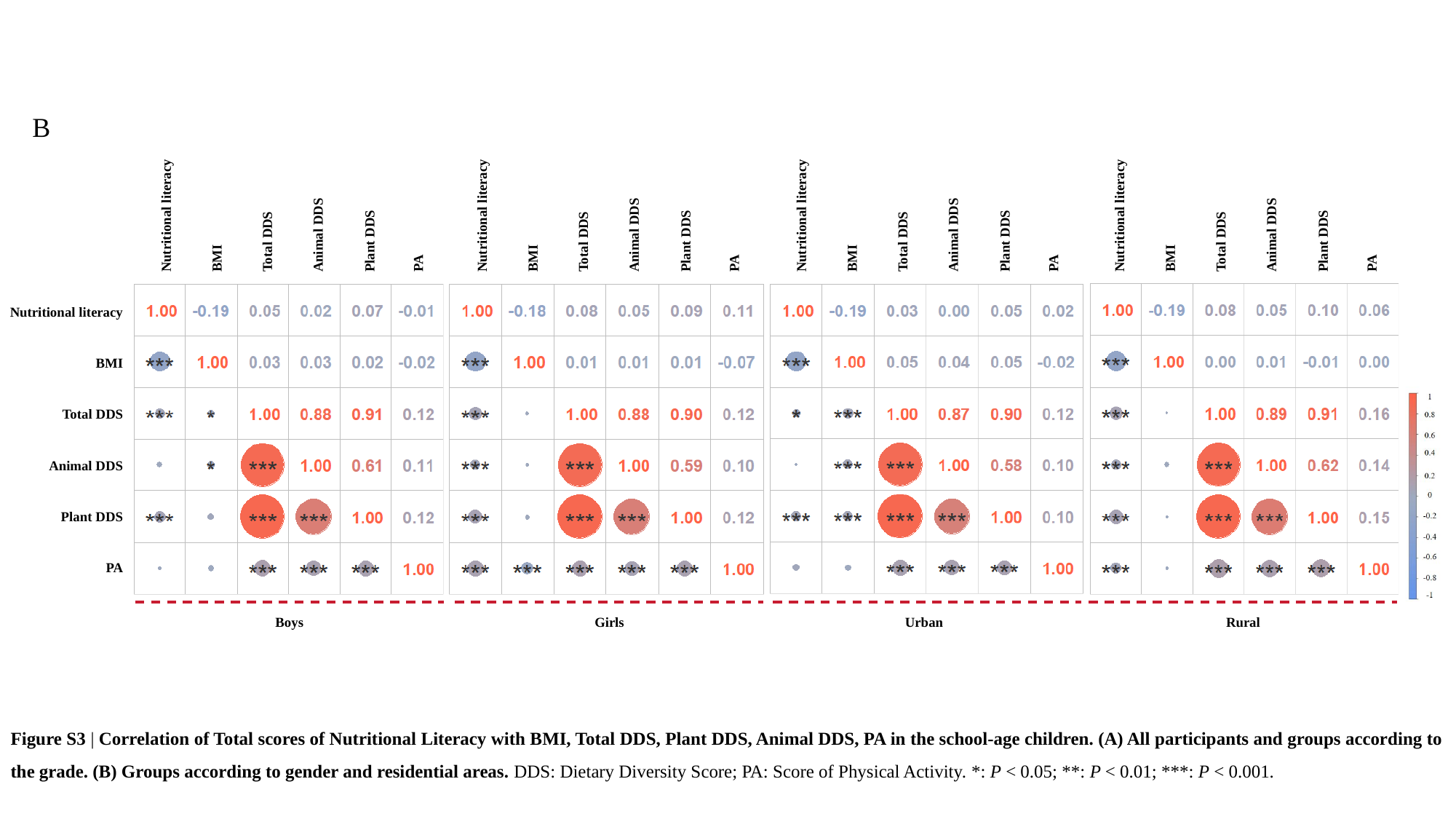

B
Nutritional literacy
BMI
Total DDS
Animal DDS
Plant DDS
PA
Nutritional literacy
BMI
Total DDS
Animal DDS
Plant DDS
PA
Nutritional literacy
BMI
Total DDS
Animal DDS
Plant DDS
PA
Nutritional literacy
BMI
Total DDS
Animal DDS
Plant DDS
PA
Nutritional literacy
BMI
Total DDS
Animal DDS
Plant DDS
PA
Boys
Girls
Urban
Rural
Figure S3 | Correlation of Total scores of Nutritional Literacy with BMI, Total DDS, Plant DDS, Animal DDS, PA in the school-age children. (A) All participants and groups according to the grade. (B) Groups according to gender and residential areas. DDS: Dietary Diversity Score; PA: Score of Physical Activity. *: P < 0.05; **: P < 0.01; ***: P < 0.001.
