# Supplementary material for: Gender-, Age-, and Region-Specific Associations Between Obesity and Nutrition/Health Knowledge, Dietary Diversity, and Physical Activity in Chinese School-Age Students: A Cross-Sectional Study
Source: Nutrients. 2025 Jul 3;17(13):2214. doi: 10.3390/nu17132214 (PMC12252032; doi:10.3390/nu17132214)
Supplement: Supplementary file 1 [file nutrients-17-02214-s001.zip › Supplementary Table.pdf]

Table S_1 Diagnostic criteria of child obesity (kg/m^2)

Age (year)	Male		Female	
	overweight	obesity	overweight	obesity
6.0-	16.4	17.7	16.2	17.5
6.5-	16.7	18.1	16.5	18.0
7.0-	17.0	18.7	16.8	18.5
7.5-	17.4	19.2	17.2	19.0
8.0-	17.8	19.7	17.6	19.4
8.5-	18.1	20.3	18.1	19.9
9.0-	18.5	20.8	18.5	20.4
9.5-	18.9	21.4	19.0	21.0
10.0-	19.2	21.9	19.5	21.5
10.5-	19.6	22.5	20.0	22.1
11.0-	19.9	23.0	20.5	22.7
11.5-	20.3	23.6	21.1	23.3
12.0-	20.7	24.1	21.5	23.9
12.5-	21.0	24.7	21.9	24.5
13.0-	21.4	25.2	22.2	25.0
13.5-	21.9	25.7	22.6	25.6
14.0-	22.3	26.1	22.8	25.9
14.5-	22.6	26.4	23.0	26.3
15.0-	22.9	26.6	23.2	26.6
15.5-	23.1	26.9	23.4	26.9
16.0-	23.3	27.1	23.6	27.1
16.5-	23.5	27.4	23.7	27.4
17.0-	23.7	27.6	23.8	27.6
17.5-	23.8	27.8	23.9	27.8
18.0-	24.0	28.0	24.0	28.0

Table S_2 Dietary frequency grading and scoring

Frequency	Level	Score
≥ 5 times/week	Frequently	2
1-4 times/week	Occasionally	1
< 1 time/week	Hardly	0

Table S_3 Physical activity grading and scoring

Frequency	Level	Score
≥ 90 minutes/day	Perfect	4
60-89 minutes/day	Frequently	3
30-59 minutes/day	Occasionally	2
< 30 minutes/day	Hardly	1

Table S_4 DDS and PA Score Grading

DDS or PA	Low	High
Total DDS	0-11	12-18
Plant DDS	0-7	8-10
Animal DDS	0-5	6-8
PA	1	2-4

Table S_5 Partial questionnaire presentation

Code: □□□□□□□□□□□□

Nutrition and Health Knowledge Survey Questionnaire for School-Age Children (Junior Primary School Edition)

Hello! This survey aims to understand your knowledge of nutrition and health. It is anonymous, and all personal information will be kept confidential. Your input is highly valuable. Thank you for your participation!

Working Group for School-Age Children's Nutrition and Health Knowledge Survey

----

Section 1: Basic Information

I. Basic Information

1. Gender: ( )

① Male ② Female

2. Date of Birth (Gregorian calendar): _____ Year _____ Month

3. School: _____

4. Class: _____ Grade _____ Class

5. Student ID: _____

6. Height: _____ cm, Weight: _____ kg (keep one decimal place)

7. Are you a boarding student? ( )

① Yes ② No

8. Do you eat at school? ( ) (Multiple choices allowed)

① No ② Breakfast (Snack) ③ Lunch ④ Dinner ⑤ Snacks

9. Who usually cooks at home? ( )

① Dad ② Mom ③ Grandparents [Grandpa/Grandma, Grandma/Grandpa (Maternal/Paternal)] ④ Myself ⑤ Housekeeper or other personnel

10. Parents' Education Level:

10.1 Father: ( )

① Primary school or below ② Junior high school ③ High school/Technical school/Vocational school ④ College/University ⑤ Bachelor's degree ⑥ Master's degree or above ⑦ Don't know

10.2 Mother: ( )

① Primary school or below ② Junior high school ③ High school/Technical school/Vocational school ④ College/University ⑤ Bachelor's degree ⑥ Master's degree or above ⑦ Don't know

11. Do you have your own bedroom? ( )

① Yes ② No

II. Nutrition and Health Knowledge

1. Please judge whether the following statements are correct. (Mark √ for correct, × for incorrect)

1.1 It's okay to skip breakfast; just eat more at lunch.

1.2 You should eat eggs or other egg products every day.

1.3 If you don't like vegetables, you can replace them with fruits.

1.4 Soybeans can be used to make tofu, bean skin, soy milk, etc.

1.5 Drinking sugary beverages can easily cause tooth decay.

1.6 Eating too many biscuits and cream cakes can easily lead to weight gain.

1.7 Eating carrots is good for protecting eyesight.

1.8 Regular sun exposure helps strengthen bones.

1.9 Fruits that look clean don't need to be washed and can be eaten directly.

1.10 Bread with mold or spots should not be eaten.

1.11 If you don't like certain foods, you don't have to eat them.

1.12 Food won't spoil if it's kept in the refrigerator.

2. Which of the following meal plates is healthier? ( )

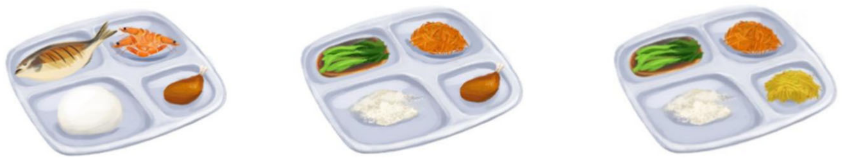

① ② ③

① Steamed fish + Fried prawns + Fried chicken leg + Steamed bun

② Stir-fried greens + Stir-fried carrot + Fried chicken leg + Rice

③ Stir-fried greens + Stir-fried carrot + Stir-fried potato + Rice

3. Which of the following beverages is recommended to drink less? ( )

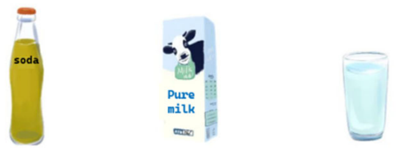

① Soda ② Milk ③ Plain water

4. In the following scenario, what is the correct behavior? ( )

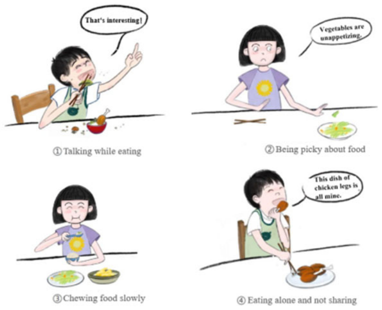

① Talking while eating ② Being picky about food

③ Chewing food slowly ④ Eating alone and not sharing

5. From the perspective of food types, select the food that does not belong to the same category in each row and mark the corresponding number with a check.

5.1

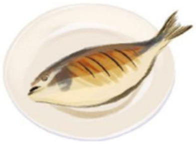
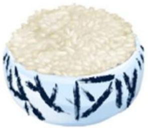
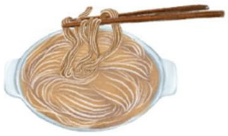

① Fish ② Rice ③ Noodles

5.2

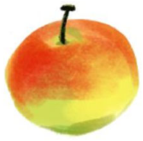
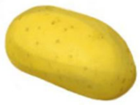
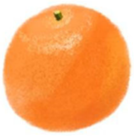

① Apple ② Potato ③ Orange

6. For health, which of the following two foods in each row is better? (Fill in the number in the parentheses after each question)

6.1 ( )

① Orange juice is better

② Orange is better

③ Both are equally good

④ Don't know

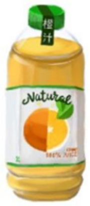

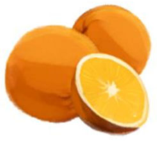

6.2 ( )

① Steamed chicken wings are better

② Fried chicken wings are better

③ Both are equally good

④ Don't know

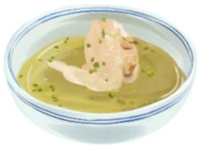

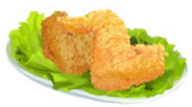

7. Which of the following foods is more suitable as a snack? ( )

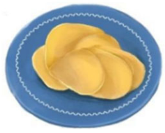

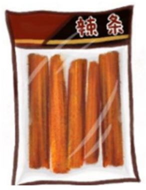

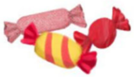

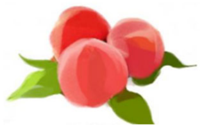

① Potato chips ② Spicy strips ③ Candy ④ Peach

III. Behavioral Section

1. How often did you eat breakfast (snack) in the past week? ( )

① Every day ② 5-6 days a week ③ 3-4 days a week ④ 1-2 days a week ⑤ Did not eat breakfast

2. In the past week, how many days did you eat the following types of food? (Mark √ in the corresponding box for each row, only one choice per row)

Category	① Daily	②5-6 days/week	③3-4 days/week	④1-2 days/week	⑤<1 day/week
2.1 Grains and tubers (rice, steamed buns, sweet potatoes, etc.)	☐	☐	☐	☐	☐
2.2 Vegetables (cucumber, beans, tomatoes, etc.)	☐	☐	☐	☐	☐
2.3 Fruits (watermelon, mango, peach, etc.)	☐	☐	☐	☐	☐
2.4 Dairy (milk, yogurt, cheese, etc.)	☐	☐	☐	☐	☐
2.5 Soy products	☐	☐	☐	☐	☐
2.6 Eggs (chicken eggs, quail eggs, etc.)	☐	☐	☐	☐	☐
2.7 Sugary beverages (cola, iced tea, etc.)	☐	☐	☐	☐	☐
2.8 Meat (beef, lamb, chicken, etc.)	☐	☐	☐	☐	☐
2.9 Coarse grains (corn, millet, mung beans, etc.)	☐	☐	☐	☐	☐
2.10 Aquatic products (fish, shrimp, shellfish, etc.)	☐	☐	☐	☐	☐

3. How much time do you spend on outdoor activities each day? ( )

(Outdoor activities refer to physical activities conducted outdoors, including outdoor physical education classes and extracurricular sports activities, walking, cycling, etc.)

① Less than 30 minutes ② 30~60 minutes ③ 60~90 minutes ④ 90~120 minutes

⑤ More than 120 minutes

Code: □□□□□□□□□□□□□

Nutrition and Health Knowledge Survey Questionnaire for School-Age Children (Senior Primary School Edition)

Hello! This survey aims to understand your knowledge of nutrition and health. It is anonymous, and all personal information will be kept confidential. Your input is highly valuable. Thank you for your participation!

Working Group for School-Age Children's Nutrition and Health Knowledge Survey

----

Section 1: Basic Information

I. Basic Information

1. Gender: ( )

① Male ② Female

2. Date of Birth (Gregorian calendar): _____ Year _____ Month

3. School: _____

4. Class: _____ Grade _____ Class

5. Student ID: _____

6. Height: _____ cm, Weight: _____ kg (keep one decimal place)

7. Are you a boarding student? ( )

① Yes ② No

8. Do you eat at school? ( ) (Multiple choices allowed)

① No ② Breakfast (Snack) ③ Lunch ④ Dinner ⑤ Snacks

9. Who usually cooks at home? ( )

① Dad ② Mom ③ Grandparents [Grandpa/Grandma, Grandma/Grandpa (Maternal/Paternal)] ④ Myself ⑤ Housekeeper or other personnel

10. Parents' Education Level:

10.1 Father: ( )

① Primary school or below ② Junior high school ③ High school/Technical school/Vocational school ④ College/University ⑤ Bachelor's degree ⑥ Master's degree or above ⑦ Don't know

10.2 Mother: ( )

① Primary school or below ② Junior high school ③ High school/Technical school/Vocational school ④ College/University ⑤ Bachelor's degree ⑥ Master's degree or above ⑦ Don't know

11. Do you have your own bedroom? ( )

① Yes ② No

Section 2: Nutrition and Health Knowledge

(I) Single-Choice Questions

1. Which food combination is the healthiest? ( )

① Oranges, chicken legs, greens, tomatoes

② Oranges, chicken legs, greens, rice

③ Bananas, beef, fish, noodles

④ Greens, tomatoes, corn, rice

⑤ Don’t know

2. Sunlight promotes the production of which nutrient? ( )

① Protein

② Fat

③ Vitamin D

④ Iron

⑤ Don’t know

3. Which nutrient-function pairing is **incorrect**? ( )

① Calcium: Promotes bone and teeth development

② Iron: Maintains normal blood production

③ Vitamin A: Provides energy

④ Vitamin C: Boosts immunity

⑤ Don’t know

4. Which product has higher protein per 100 ml? ( )

Product 1		
Nutrition Facts (per 100ml)		
Item	Per 100ml	NRV%
Energy	309KJ	4%
Protein	3.6g	6%
Fat	4.4g	7%
Carbohydrates	5.0g	2%
Sodium	58mg	3%

Product 2		
Nutrition Facts (per 200ml)		
Item	Per serving	NRV%
Energy	542KJ	6%
Protein	6.2g	10%
Fat	7.2g	12%
Carbohydrates	10g	3%
Sodium	90mg	5%

① Product 1 ② Product 2 ③ Don’t know

5. Today is May 17, 2025. Which bread should not be eaten? ( )

① Production date: May 15, 2025; Expiry: 3 days

② Production date: May 5, 2025; Expiry: 5 days

③ Don’t know

6. Which option meets the daily recommended fruit intake (200–350g)? ( )

① Six fresh jujubes

② One medium banana

③ One kiwi

④ One orange and one medium apple

⑤ Don’t know

7. Where should these foods be stored? (Check ✓ one box per row.)

	① Cool & Ventilated	② Refrigerator	③ Freezer
7.1 Raw meat (not eaten today)	☐	☐	☐
7.2 Cut watermelon	☐	☐	☐
7.3 Flour	☐	☐	☐
7.4 Leftovers	☐	☐	☐

(II) Multiple-Choice Questions

8. Which statements about breakfast are **incorrect**? ( )

① Skipping breakfast is fine if you eat more at lunch

② Bread and milk make a balanced breakfast

③ Soy milk + eggs + beef buns + cucumber salad is balanced

④ Eating breakfast while walking is efficient

⑤ Don't know

9. Which statements about drinking water are **correct**? (   )

① Only drink when thirsty

② Drink actively and frequently in small amounts

③ Sugary drinks cannot replace water

④ Thirst indicates dehydration

⑤ Don't know

10. Which statements are **incorrect**? (   )

① Eating speed has little impact on health

② Watching TV while eating is unhealthy

③ Skip disliked foods

④ Overeat favorite foods even when full

⑤ Don't know

11. Which statements about Chinese dietary culture are **correct**? (   )

① Many regions have unique local cuisines

② Dumplings, mooncakes, and yuanxiao are traditional foods

③ Eating zongzi is a Qingming Festival tradition

④ Not speaking with food in the mouth is part of dining etiquette

⑤ Don't know

12. Which food-crop pairings are **correct**? (   )

① Potato chips – potatoes

② Steamed buns – wheat

③ Rice – rice plants

④ Fish tofu – soybeans

⑤ Don't know

13. Which statements are **correct**? (   )

① Sugary drinks cause tooth decay

② Sugary drinks contribute to weight gain

③ Long-term "zero-sugar" drinks are harmless

④ Sweets provide energy for growth; eat more

⑤ Don’t know

14. Which statements are **correct**? (   )

① Excess salt increases hypertension risk

② Vegetable oil is healthier; eat more

③ Excess cooking oil causes obesity

④ Children won’t get hypertension; eat more salt

⑤ Don’t know

15. Which statements are **correct**? (   )

① Dark vegetables like carrots protect eyesight

② Moderate meat intake builds muscle

③ Sunlight strengthens bones

④ Whole grains improve gut health

⑤ Don’t know

16. Which practices maintain a healthy weight? (   )

① Avoid rice/noodles

② Exercise daily

③ Eat little/no dinner

④ Limit high-fat/sugar foods

⑤ Don’t know

17. Which actions reduce food waste? (   )

① Stockpile a week’s groceries in the fridge

② Ask cafeteria staff to serve smaller portions

③ Order freely at restaurants without considering waste

④ Take leftovers home

⑤ Don’t know

18. Which foods are high in oil/salt? (   )

① Salted walnuts

② Potato chips

③ Spicy strips

④ Yogurt

⑤ Don't know

19. Which statements about dining out are **correct**? (   )

① Request less oil/salt

② Restaurant food often has excess oil/salt

③ Avoid fried foods like chicken wings

④ Eat less staple food when dining out

⑤ Don't know

20. Which statements are **correct**? (   )

① Reheat leftovers thoroughly

② Freshly picked fruits can be eaten unwashed

③ Wash hands before cooking

④ Use serving utensils at home/restaurants

⑤ Don't know

21. Which statements are **correct**? (   )

① Cut moldy parts off apples; eat the rest

② Moldy bread is unsafe

③ Avoid picking/eating colorful wild mushrooms

④ Avoid eating wild animals

⑤ Don't know

(III) Matching Questions

22. Match food categories to their layers in the Chinese Dietary Guidelines Pagoda (5 layers, varying sizes indicate recommended intake):

22.1 Fish, poultry, eggs, lean meat

22.2 Oil and salt

22.3 Grains and tubers (rice, noodles, potatoes)

22.4 Dairy (milk, yogurt) and soy products (tofu, soy milk)

22.5 Vegetables and fruits

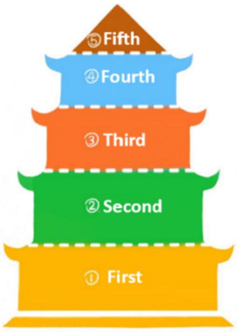

23. Match foods to their categories:

23.1 Corn ① Vegetables

23.2 Banana ② Grains/Tubers

23.3 Eggplant ③ Fruits

24. Match foods to their primary nutrients:

24.1 Beef ① Iron

24.2 Animal liver ② Vitamin C

24.3 Rice ③ Protein

24.4 Fresh jujube ④ Carbohydrates

Section 3: Behavioral Habits

1. How often did you eat breakfast last week? (   )

① Daily

② 5 – 6 days/week

③ 3 – 4 days/week

④ 1 – 2 days/week

⑤ Never

2. Frequency of consuming these foods last week: (Check ✓ one box per row.)

Category	① Daily	②5-6 days/week	③3-4 days/week	④1-2 days/week	⑤<1 day/week
2.1 Grains and tubers (rice, steamed buns, sweet potatoes, etc.)	☐	☐	☐	☐	☐
2.2 Vegetables (cucumber, beans, tomatoes, etc.)	☐	☐	☐	☐	☐
2.3 Fruits (watermelon, mango, peach, etc.)	☐	☐	☐	☐	☐
2.4 Dairy (milk, yogurt, cheese, etc.)	☐	☐	☐	☐	☐
2.5 Soy products	☐	☐	☐	☐	☐
2.6 Eggs (chicken eggs, quail eggs, etc.)	☐	☐	☐	☐	☐
2.7 Sugary beverages (cola, iced tea, etc.)	☐	☐	☐	☐	☐
2.8 Meat (beef, lamb, chicken, etc.)	☐	☐	☐	☐	☐
2.9 Coarse grains (corn, millet, mung beans, etc.)	☐	☐	☐	☐	☐
2.10 Aquatic products (fish, shrimp, shellfish, etc.)	☐	☐	☐	☐	☐

3. Daily **outdoor** activity duration? (   )

① <30 minutes

② 30 – 60 minutes

③ 60 – 90 minutes

④ 90 – 120 minutes

⑤ >120 minutes
